# Population Selection and Sequencing of *Caenorhabditis elegans* Wild Isolates Identifies a Region on Chromosome III Affecting Starvation Resistance

**DOI:** 10.1534/g3.119.400617

**Published:** 2019-08-23

**Authors:** Amy K. Webster, Anthony Hung, Brad T. Moore, Ryan Guzman, James M. Jordan, Rebecca E. W. Kaplan, Jonathan D. Hibshman, Robyn E. Tanny, Daniel E. Cook, Erik Andersen, L. Ryan Baugh

**Affiliations:** * Department of Biology, Duke University, Durham, NC; † University Program in Genetics and Genomics, Duke University, Durham, NC, and; ‡ Department of Molecular Biosciences, Northwestern University, Evanston, IL

**Keywords:** L1 arrest, starvation resistance, diapause, population sequencing, GWAS, RAD-seq

## Abstract

To understand the genetic basis of complex traits, it is important to be able to efficiently phenotype many genetically distinct individuals. In the nematode *Caenorhabditis elegans*, individuals have been isolated from diverse populations around the globe and whole-genome sequenced. As a result, hundreds of wild strains with known genome sequences can be used for genome-wide association studies (GWAS). However, phenotypic analysis of these strains can be laborious, particularly for quantitative traits requiring multiple measurements per strain. Starvation resistance is likely a fitness-proximal trait for nematodes, and it is related to metabolic disease risk in humans. However, natural variation in *C. elegans* starvation resistance has not been systematically characterized, and precise measurement of the trait is time-intensive. Here, we developed a population-selection-and-sequencing-based approach to phenotype starvation resistance in a pool of 96 wild strains. We used restriction site-associated DNA sequencing (RAD-seq) to infer the frequency of each strain among survivors in a mixed culture over time during starvation. We used manual starvation survival assays to validate the trait data, confirming that strains that increased in frequency over time are starvation-resistant relative to strains that decreased in frequency. Further, we found that variation in starvation resistance is significantly associated with variation at a region on chromosome III. Using a near-isogenic line (NIL), we showed the importance of this genomic interval for starvation resistance. This study demonstrates the feasibility of using population selection and sequencing in an animal model for phenotypic analysis of quantitative traits, documents natural variation of starvation resistance in *C. elegans*, and identifies a genomic region that contributes to such variation.

In order to determine the genetic basis of quantitative traits, it is important to efficiently phenotype many individuals across many genotypes. By using panels of isogenic lines from a model organism of interest, controlled experiments can be performed for particular traits, and biological replicates facilitate repeat measurements of the same genotype. There are a number of model organisms for which panels of wild isogenic lines have been generated and sequenced, including the roundworm *Caenorhabditis elegans* (Churchill *et al.* 2004; Mackay *et al.* 2012; Weigel 2012; Cook *et al.* 2017; Noble *et al.* 2017; Srivastava *et al.* 2017; Peter *et al.* 2018). *C. elegans* is particularly well-suited to investigating natural variation of life-history traits, as it reproduces predominantly through selfing and is mostly homozygous upon collection. It does not suffer from inbreeding depression (Dolgin *et al.* 2007; Chelo *et al.* 2014), which can be problematic for many species (Radwan 2003; Shorter *et al.* 2017). A single wild worm can be used to generate an isogenic line. Lines can also be stored long-term at -80° and thawed as needed to avoid drift or inadvertent selection. Hundreds of *C. elegans* wild isolates have been collected from around the globe, whole-genome sequenced and made available for use through the *Caenorhabditis elegans* Natural Diversity Resource (CeNDR) (Cook *et al.* 2017; Hahnel *et al.* 2018). These lines represent the natural diversity of the species, rather than being generated through mutagenesis (Jorgensen and Mango 2002), experimental mutation accumulation (Denver *et al.* 2004; Denver *et al.* 2009), or recombination of multiple parental strains (Noble *et al.* 2017). As a result, differences in traits across these strains suggest variation in these traits in the wild. Further, phenotypically divergent wild strains can be used to make recombinant strains in order to fine map genetic variants.

Progression through the *C. elegans* life cycle depends on food availability. In rich conditions, larvae develop through four larval stages and become reproductive adults in a couple of days. However, *C. elegans* larvae display developmental plasticity and arrest development in dauer diapause as an alternative to the third larval stage (L3) in response to high population density, limited nutrient availability, or high temperature (Riddle and Albert 1997). Animals can survive dauer diapause for months and resume reproductive development if conditions improve. In a related but distinct phenomenon, L1 larvae that hatch in the complete absence of food (*E. coli* in the lab) arrest development in a state known as “L1 arrest” or “L1 diapause” (Baugh 2013). Larvae can survive starvation during L1 arrest for weeks, and arrest is reversible upon feeding. Larvae can also arrest development at later stages in response to acute starvation (Schindler *et al.* 2014), and adults arrest reproduction in response to starvation (Angelo and Van Gilst 2009; Seidel and Kimble 2011). It is relatively straight-forward to produce pure populations of larvae in L1 arrest, facilitating investigation of the starvation response. Notably, *Caenorhabditis* are frequently found in a starved state in the wild (Barriere and Felix 2006), reflecting the importance of investigating starvation resistance to characterize variation in the wild.

In terms of organismal fitness, it is important to not only *survive* starvation but to also *recover* from it and ultimately produce progeny. As a result, when we refer to “starvation resistance”, we consider three key facets, including survival, growth rate following starvation, and progeny production following starvation, each of which is affected by L1 starvation. Differences in starvation survival are often coupled to differences in recovery and fecundity (Jobson *et al.* 2015; Webster *et al.* 2018), but these traits can also be uncoupled in the context of particular genetic or environmental perturbations (Kaplan *et al.* 2015; Hibshman *et al.* 2016; Roux *et al.* 2016). Effects on survival, growth rate and fecundity are each integral to the effect of L1 starvation on fitness.

In addition to its significance as a determinant of evolutionary fitness, the genetic basis of starvation resistance is of particular interest for its relevance to aging, cancer, diabetes, and obesity. L1 arrest and dauer diapause have been termed “ageless” states because length of time spent in arrest has no effect on the duration of adult lifespan after recovery (Klass and Hirsh 1976; Johnson *et al.* 1984), though larvae in L1 arrest display signs of aging, most of which are reversed upon recovery (Roux *et al.* 2016). In addition, pathways that affect starvation resistance in larvae typically affect aging in adults (Baugh 2013). Furthermore, insulin/IGF signaling and AMP-activated protein kinase (AMPK) affect starvation resistance in worms and are conserved in mammals, where they are implicated in diabetes and cancer (Malaguarnera and Belfiore 2011; Li *et al.* 2015). Genetic analysis of *C. elegans* has historically been performed in the genetic background of the commonly used lab strain, N2. Consequently, prior studies have not leveraged the natural variation of starvation resistance among wild isolates to identify genetic variants that impact this trait. We seek to determine how much variation in starvation resistance is present across wild strains, identifying strains that are particularly sensitive and resistant to starvation. This will position us to identify genetic variants associated with differences in starvation resistance both 1) to understand the architecture of this important trait and 2) to identify particular genes and variants that affect it in wild populations.

Here, we used a panel of 96 whole-genome-sequenced wild strains to determine the extent of natural variation in starvation resistance with a population-selection-and-sequencing approach that is novel for animal models. We pooled and starved the strains in a single culture, and then used DNA sequencing to infer the relative frequency of survivors over time after recovery from starvation, incorporating the three facets of starvation resistance into a single assay. In theory, with a single culture and multiplexed measurements, technical variation is reduced as all strains share a common environment. We identified strains that increased or decreased the most in frequency using RAD-seq (Andrews *et al.* 2016) on populations, and we inferred a trait value for starvation resistance for each strain. We confirmed that the most divergent strains are starvation-resistant or sensitive using traditional assays. We used a GWAS to identify a region on chromosome III significantly associated with increased starvation resistance, and we validated this association with near-isogenic lines. This work demonstrates that population-selection-and-sequencing approaches can be used in *C. elegans* to efficiently phenotype many strains with adequate precision for GWAS. Further, we show that there is natural variation in starvation resistance in *C. elegans*, and a region on chromosome III explains some of that variation.

## Materials and Methods

### Strains

Ninety-six *C. elegans* wild isolate strains were used for pooling in the RAD-seq experiment, including AB1, AB4, CB4851, CB4852, CB4853, CB4854, CB4856, CB4857, CB4858, CB4932, CX11262, CX11264, CX11271, CX11285, CX11292, CX11307, CX11314, CX11315, DL200, DL226, DL238, ED3005, ED3011, ED3012, ED3017, ED3040, ED3046, ED3048, ED3049, ED3052, ED3073, ED3077, EG4347, EG4349, EG4724, EG4725, EG4946, JT11398, JU1088, JU1172, JU1200, JU1212, JU1213, JU1242, JU1246, JU1395, JU1400, JU1409, JU1440, JU1491, JU1530, JU1568, JU1580, JU1581, JU1586, JU1652, JU1896, JU258, JU310, JU311, JU323, JU346, JU360, JU363, JU367, JU393, JU394, JU397, JU406, JU440, JU561, JU642, JU751, JU774, JU775, JU778, JU782, JU792, JU830, JU847, KR314, LKC34, LSJ1, MY1, MY10, MY16, MY18, MY23, PB303, PB306, PS2025, PX174, PX179, QX1211, QX1233, WN2002. These strains were obtained from the *Caenorhabditis* Genetics Center (CGC). In addition, Bristol N2 was used in validation experiments, along with QX1430, a modified N2 strain that is genetically compatible with strains that lack *peel-1**/**zeel-1* (Andersen *et al.* 2015). LRB361 and LRB362 were generated to validate the chromosome III interval.

### Worm culture and sample collection for RAD-seq

Ninety-six strains were maintained on *E. coli* OP50-seeded nematode growth medium (NGM) plates at 20°. All strains were maintained well-fed for at least three generations prior to experiments to control for intergenerational or transgenerational effects of starvation (Jobson *et al.* 2015; Hibshman *et al.* 2016; Webster *et al.* 2018; Jordan *et al.* 2019). Larvae were washed from clean, starved 6 cm plates in parallel with S-complete, one plate per strain, and pooled. 20% of this pool was cultured in liquid with 50 mg/mL *E. coli* HB101 in 500 mL total volume of S-complete. After 67 - 72 hr at 20° and 180 rpm, this culture was hypochlorite treated to obtain an axenic population of embryos, with a yield of 7 - 14 million embryos. Embryos were resuspended at a density of 10 per µL in virgin S-basal (no ethanol or cholesterol). These embryos hatched and entered L1 arrest. For the first biological replicate, two time points were sampled at days 16 and 21 (early time points were sampled, and survivors were separated from dead worms with a sucrose float, but this approach did not work well in later time points, and these samples were abandoned). For the second biological replicate, the culture was sampled at days 1, 7, 14, 21, and 24. For sampling, survival was scored, and approximately 1500 survivors were plated per 10 cm NGM plate with an OP50 lawn (larger volumes of the starvation culture were sampled in later time points as survival decreased; worms were pelleted by centrifuge and plated in 1 mL), with ten plates per time point. Survival was 49.3% at day 16 for replicate 1, and 48.6% for day 14 for replicate 2. Recovery plates were cultured until all OP50 on the plate was consumed. This initially took about four days, but in the later time points additional time was necessary. This duration allows survivors to recover, grow, and begin to produce progeny. Starved recovery plates were washed with virgin S-basal. Cultures were spun down at 3000 rpm for 1 min and excess S-basal was aspirated off. Worms were pipetted into a 1.5 mL tube and flash frozen with liquid nitrogen prior to storage at -80°. The Qiagen DNAeasy Blood and Tissue column-based kit (Catalog #69504) was used to prepare genomic DNA.

### Restriction site-associated DNA sequencing (RAD-seq)

RAD-seq was performed as previously described (Davey and Blaxter 2010) in the Duke GCB Sequencing and Genomic Technologies Shared Resource. DNA from each timepoint was digested with *Eco*RI, and adapters were ligated. The ligation products were sheared, and a second adapter was ligated. This second adapter cannot bind to a PCR primer unless its sequence is completed by the amplification starting from a primer bound to the first adapter. This selectively amplified only fragments containing both adapters. The fragments were amplified through PCR and then size selected for 200-500 bp fragments. These fragments were sequenced on an Illumina HiSeq 2500 producing 50 base pair single-end reads. All sequencing data are archived at NCBI under BioProject number PRJNA533789. The 96 strains used were previously sequenced by RAD-seq, and SNVs were called (Andersen *et al.* 2012).

### Data processing

RAD-seq 50 base-pair FASTQ reads were mapped to version WS210 of the *C. elegans* genome using version 0.7.4 of bwa with default parameters (Li and Durbin 2009). SNVs were called using the command: bcftools call -mv -0z (Li 2011). For each of the seven time points across two replicates, the total number of reads ranged from 49,116,876 to 123,890,427. For each variant, counts were stored and are available as part of Supplementary File 1. These variants were then subsequently filtered to include only unique variants. There were 12,285 total unique SNVs across all strains, with a median of 33 unique SNVs per strain (Table 1). The median read coverage per unique SNV across all strains was 1,691 (Table 1). Counts for all unique SNVs are available as part of Supplementary File 1.

**Table 1:** Strain data for 96 strains included in the RAD-seq population-sequencing experiments. The number of unique SNVs for each strain is reported, along with the average coverage per SNV and the standard error of coverage across all unique SNVs and all replicates. The RAD-seq trait value, used for the GWAS of starvation resistance, is also reported

Strain	Number of Unique SNVs	Avg Coverage per SNV	Std Error of Coverage	RAD-seq Trait Value
AB1	22	1910	132.1	−0.001089558
AB4	43	812	51.8	−0.000735581
CB4851	22	1800	165.7	−1.28E-05
CB4852	16	1564	133.9	−9.77E-05
CB4853	22	1527	114.5	−0.000104916
CB4854	182	1829	74.7	−0.000834588
CB4856	874	1915	32.6	−0.000926858
CB4857	47	1317	90.2	−0.000486995
CB4858	14	1054	92.5	−0.000279677
CB4932	31	1477	105	8.25E-05
CX11262	16	1315	107.1	−0.000776708
CX11264	29	2002	164.5	−0.001023059
CX11271	42	1884	126.2	−0.000285448
CX11285	72	2227	120.1	−0.000454118
CX11292	1	1398	316.6	0.000365799
CX11307	62	8415	2592.2	−0.000483986
CX11314	11	1612	192.5	−0.000528437
CX11315	47	1768	101.7	0.001110572
DL200	23	1446	102.6	0.005747001
DL226	117	2484	296.5	−0.000764146
DL238	966	2561	170.8	−0.000804199
ED3005	14	1789	144.3	0.001394962
ED3011	17	1296	120	−0.000241451
ED3012	16	1434	128	−0.000347688
ED3017	24	1631	129.5	0.002837005
ED3040	43	1749	121.8	−2.54E-05
ED3046	42	1779	123.5	−0.000153265
ED3048	32	1210	113.9	0.000259088
ED3049	2	979	251.8	−0.000428888
ED3052	34	1798	144.1	0.002854565
ED3073	43	1414	83.1	−0.000480596
ED3077	30	1255	75.1	0.015852705
EG4347	8	1732	309.9	0.000265527
EG4349	73	1768	103.7	0.000949259
EG4724	68	1465	72.8	−0.000240064
EG4725	213	2274	74.3	0.000711111
EG4946	25	1577	133.7	−0.001189646
JT11398	67	2661	319	−0.000251866
JU1088	38	1350	89.6	−0.000542719
JU1172	39	1875	133.9	−0.000573628
JU1200	18	1292	109.5	0.000643843
JU1212	67	2601	156.2	−0.000501071
JU1213	47	2065	110.2	0.00010362
JU1242	29	1790	145	−0.000508404
JU1246	19	1109	87.4	−0.000352744
JU1395	14	2068	239.2	−0.000812441
JU1400	66	2271	137	−9.72E-05
JU1409	73	1419	71.7	−0.000496307
JU1440	26	1452	97.7	−0.00046671
JU1491	43	1759	112.5	−0.00018339
JU1530	14	1211	109.1	−0.000394825
JU1568	28	1721	130.7	−9.76E-06
JU1580	28	1586	107.8	0.000636567
JU1581	67	1591	103.4	−0.001178581
JU1586	22	1228	89.8	−0.000505315
JU1652	77	1682	97.2	0.004598108
JU1896	121	1825	69.9	0.001495203
JU258	147	2293	102.4	−0.000978538
JU310	20	1642	133.8	−0.000783759
JU311	25	1678	138.8	−0.000460506
JU323	35	14009	4563.5	−0.000451522
JU346	45	2322	201.2	−0.000320575
JU360	9	1163	145	−0.000889179
JU363	5	1188	168.9	0.000173825
JU367	24	1497	190.5	−0.000317346
JU393	20	1877	179.3	−0.00097683
JU394	27	1700	115	8.55E-05
JU397	21	1291	90.2	−1.14E-05
JU406	19	1466	158.3	7.70E-05
JU440	24	1408	105.4	−0.000588908
JU561	42	1306	72.1	−0.000947311
JU642	27	1418	109.4	−0.000175764
JU751	19	2271	243.7	−0.000343356
JU774	87	2017	120.3	0.000156211
JU775	726	1794	30.7	−0.00013356
JU778	91	1430	54.9	−0.000466356
JU782	100	2863	152.5	−0.000251516
JU792	26	1424	125.5	−0.000126172
JU830	22	1760	187.8	−0.000421824
JU847	33	1529	101.4	0.000694595
KR314	83	1789	73.6	−0.000739883
LKC34	58	1484	82.4	0.000101218
LSJ1	7	1962	341.3	0.000228846
MY1	39	1992	143.8	−0.000355746
MY10	148	3028	106.3	−0.000267622
MY16	41	1743	124.6	−0.001084975
MY18	26	14878	6112.5	0.000116325
MY23	176	1821	68	−0.000536114
PB303	16	1354	143.8	−0.001618959
PB306	220	2071	69.7	−0.000679825
PS2025	99	2002	122	−0.002479676
PX174	46	1302	86.2	−0.000170046
PX179	13	1289	108.7	−0.000586876
QX1211	5355	2407	35.8	−0.000186515
QX1233	53	1493	91.3	−0.000967571
WN2002	65	1442	110.1	−0.000671692

### Theoretical error estimation for strain frequency

We determined the theoretical error of strain frequency estimates obtained from RAD-seq data given a median read coverage per SNV of 1,691 (Supplementary Figure 1). We used the rpois function in R for the Poisson distribution to simulate the number of alternative allele (strain-specific) reads for a unique SNV as a function of strain frequency. We varied both the expected number of reads mapping to the unique SNV (used to determine a particular strain’s frequency based on the 1,691 total reads in the real data) and the number of unique SNVs (since this varies across strains). To simulate multiple SNVs, we drew from the Poisson distribution multiple times and averaged those estimates, mimicking how we determined a strain’s frequency using the RAD-seq data. We then determined the error between the estimated frequency from simulation with the Poisson distribution as the absolute difference from the theoretical frequency. We determined the average proportional error by taking the average absolute difference between the estimated and theoretical frequencies and dividing by the theoretical frequency. The lines plotted in Supplementary Figure 1 are the average of 4,000 simulations.

### Counts to trait values for GWAS

Count tables were filtered to only include counts for unique SNVs previously identified (Andersen *et al.* 2012). The count table for unique SNVs is available as part of Supplementary File 1. Unique SNVs are those for which only one strain out of the 96 has the alternative SNV. For each strain and library/timepoint, the average alternative SNV frequency was calculated, approximating the frequency of each strain. For biological replicates one and two, linear regressions were calculated with day as the independent variable and the average value for the strain as the dependent variable. The slopes of these regressions indicate whether the representation of a strain tends to increase or decrease over time in culture. The slopes of these linear regressions for replicates one and two were averaged, and these “RAD-seq trait values” were used for GWAS (Table 1). The R script to go from unique SNV count values to trait values used for GWAS is available at github.com/amykwebster/RADseq_2019_StarvationResistance

### GWAS using CeNDR

The *Caenorhabditis elegans* Natural Diversity Resource (CeNDR) was used to perform GWAS (Cook *et al.* 2017) using the EMMA algorithm via the rrBLUP package (Kang *et al.* 2008; Endelman 2011). The RAD-seq trait values and strain names were used as input for GWAS. The CeNDR version used was 1.2.9, with data release 20180527 and cegwas version 1.01. Version WS263 of the worm genome was used in this data release. Data for strains CB4858 and JU363 were removed to run GWAS because these were considered to be from the same isotype on CeNDR.

### L1 starvation survival

Strains of interest were cultured on 10 cm NGM plates seeded with *E. coli* OP50. Gravid adults were hypochlorite treated to obtain axenic embryos. Embryos were re-suspended in virgin S-basal at a density of 1/µL and cultured so they hatch and enter L1 arrest. A 5 mL volume was used in a 16 mm glass test tube with a plastic cap, which was incubated on a tissue culture roller drum at ∼21.5° (room temperature) for the duration of the experiment.

Beginning on day 1 and continuing every other day, 100 µL samples were taken from the L1 arrest culture and plated on a 5 cm NGM plate with a spot of OP50 in the center. The L1 larvae were plated around the outside of the spot of OP50. The number of worms plated (T_p_) was counted. Two days later, the number of live worms was counted (T_a_). Live worms are considered those that were able to crawl to the OP50 to begin feeding. The rest of the plate was also scanned to include worms that had begun to develop but were not on the patch of OP50. The percent survival on each day was calculated as total alive divided by total plated (T_a_/T_p_). For Figure 2A, four biological replicates were scored for seven strains (N2, CB4856, DL200, ED3077, JU1652, JU258, JU561) and three biological replicates were scored for one strain (QX1430). For Figure 4A, four to five biological replicates were scored for each of the four strains. Logistic curves were fit to each biological replicate and strain in R using the package ‘wormsurvival’ (Kaplan *et al.* 2015), and the median survival time was calculated. Bartlett’s test was used to test for differences in variances among median survival times across strains. For instances in which there were no significant differences in variance, we pooled the variances across strains for further analysis. We performed unpaired *t*-tests for comparisons of interest.

### Total brood size

For total brood size (Figure 2C), worms starved as L1 larvae for one day or eight days (as described under ‘L1 Starvation Survival’) were plated on 5 cm NGM plates with OP50 and allowed to recover for 48 hr at 20°. After 48 hr, worms were singled onto plates with OP50. Every 24 hr, the individual worms were each transferred to a new plate until egg laying ceased (96 hr). 48 hr after eggs were laid, the number of progeny on each plate was scored. The total brood size for each worm was calculated as the total number of progeny laid on all plates for a single worm. 1,152 total individual worms were scored, including eighteen individual worms for each strain (N2, CB4856, JU561, ED3077), time starved (one or eight days), and biological replicate (eight biological replicates). No worms were censored from analysis, including those that died during egg laying or produced no progeny. For statistical analysis, the R package ‘nlme’ was used to fit linear mixed-effects models to the data. For each strain, the number of days of starvation (one or eight) was a fixed effect, and biological replicate was a random effect. To test for interactions between days of starvation and strain, we included both genotype and days of starvation as fixed effects, and included an interaction term in the model. To assign a p-value, we used the “summary” function in R, which performed a *t*-test on the t-value. For significant p-values, we rejected the null hypothesis that the slope of the linear mixed-effects model for the variable of interest was zero.

### Starvation recovery and early fecundity

L1 larvae were starved for one or eight days (as described under ‘L1 Starvation Survival’). At each time point, larvae were plated on NGM plates with OP50 bacteria and allowed to recover for 48 hr at 20°. To measure worm body length, after 48 hr of recovery, worms were washed off the recovery plates with virgin S-basal and plated on unseeded 10 cm NGM plates for imaging. Images were taken on a ZeissDiscovery V20 stereomicroscope. Images were analyzed with the WormSizer plugin for FIJI to determine worm length and manually passed or failed (Moore *et al.* 2013). For early fecundity, worms that recovered for 48 hr were singled onto plates with OP50 and allowed to lay embryos for 24 hr and were then removed. After two days, the progeny on the plates were counted. For both body length and early fecundity, measurements after eight days of starvation were normalized by dividing by the average for the same strain after only one day of starvation. Statistics were performed on these normalized values, and a linear mixed-effects model was implemented using the ‘nlme’ package in R. Strain was a fixed effect and biological replicate was a random effect. For early fecundity, three biological replicates were scored, and eighteen individual worms were scored per replicate per strain. For body length following starvation and recovery, a total of 1,063 worms were included in analysis, including 48 - 82 individual worms measured per replicate and strain following one day of starvation and 8 - 68 individual worms measured for each replicate and strain following eight days of starvation. Lower numbers of individual worms following eight days of starvation were due to the effect of lethality in JU258, as only live worms were scored. Three biological replicates were scored for both assays.

### Generation of near isogenic lines (NILs)

The GWAS led to identification of a significant quantitative trait locus (QTL) located at III:292,594 associated with starvation resistance, and the linked interval consisted of III:86,993-925,945. A starvation-resistant strain, DL200, that has the alternative SNV associated with increased starvation resistance, and a starvation-sensitive strain, JU258, that has the reference SNV at this locus, were chosen to generate NILs. These strains are theoretically genetically compatible at *peel-1*/*zeel-1* and *pha-1**/**sup-35* loci (Seidel *et al.* 2008; Ben-David *et al.* 2017). Two strains were generated: one with the JU258 interval on chromosome III but with the DL200 background (JU258 > DL200, LRB362), and one with the DL200 interval but with a majority-JU258 background (DL200 > JU258, LRB361).

We identified sequences on both ends of the chromosome III interval that allowed us to differentiate between JU258 and DL200 backgrounds via restriction fragment length polymorphism. Primers were used that amplified near each end of the III:86993-925945 interval followed by restriction digest and gel electrophoresis. The primer sequences were generated using the VCF-kit tool “primer snip” for the region of interest (Cook and Andersen 2017). Primers were tested, and working primers for each end of the interval were used for genotyping. PCR was performed using *Taq* DNA polymerase. Both ends of the interval were genotyped to ensure that a recombination event did not break up the interval. The primers amplified in both DL200 and JU258 backgrounds, but the amplified region contains a SNV affecting a restriction site in one genotype but not the other. These primers are: 1) III:83270_F1 (ttggggtactgtagtcggtg), 2) III:83270_R1 (AAGCTCCTTCCACACGTACG), 3) III:729215_F1 (CGTTTGGCACGTACTGAAGC), and 4) III:729215_R3 (AGAACGTCGTAGCCGTCATC). III:83270_F1 and III:83270_R1 function as a primer pair, and *BstUI* is used to digest the PCR products. The PCR product for both DL200 and JU258 is 610 bp. When *BstUI* is used, it cuts the JU258 PCR product into 348 and 262 bp products, but it does not cut the DL200 product. III:729215_F1 and III_729215_R3 function as a primer pair, and *MseI* is used to digest the PCR products. For both JU258 and DL200, the PCR product is 700 bp. Upon digestion, the JU258 product is cut into 118 bp and 582 bp, and the DL200 product is cut into 118, 143, and 439 bp. For both enzymes, digests were performed at 37° for at least one hour.

The PCR protocol used was:

1. 95° for 30 sec
2. 95° for 15 sec
3. 55° for 30 sec
4. 68° for 1 min 20 sec
5. 68° for 5 min
6. Repeat steps 2 - 4 30x
7. Hold at 15°

To generate NILs, DL200 and JU258 worms were crossed to generate heterozygotes, which then self-fertilized to produce F2 progeny. F2 generation worms were singled and genotyped on both sides of the chromosome III peak following egg laying. For this first cross, homozygotes were chosen from both genotypes to continue crosses. From this step onward, separate crosses were performed for each genotype. Worms that were genotyped as homozygous for DL200 were crossed to JU258 males, and F2 progeny that were homozygous at the interval for DL200 were chosen again following genotyping. These were successively backcrossed to JU258 males, with homozygotes for DL200 chosen in the F2 generation each time. In total, six crosses were performed, such that the DL200 interval should be in a background consisting mostly of the JU258 genome. For the JU258 interval, homozygotes were successively backcrossed to DL200 males, with JU258 homozygotes at the chromosome III interval chosen each F2 generation. Again, six crosses were performed to generate this strain. After these crosses and genotyping, each strain was self-fertilized for six generations to ensure homozygosity. After generation of NILs, resulting strains and parent strains were whole-genome sequenced at low coverage. All sequencing data are archived on NCBI under BioProject number PRJNA533789. The VCF-kit tool “hmm”, which uses a Hidden Markov Model to infer from which parent a particular genomic region was inherited, was used to determine the sequence of the resulting strains (Cook and Andersen 2017).

### Data availability

Raw sequencing data are available through NCBI under BioProject number PRJNA533789. Files to generate trait data for GWAS are available at github.com/amykwebster/RADseq_2019_StarvationResistance, and output from GWAS is available at elegansvariation.org/report/301dcc3f/RADseq_TraitValue. Input and output of GWAS is also available as part of Supplementary File 1. Supplemental material available at FigShare: https://doi.org/10.25387/g3.9458072.

## Results

### Population selection and sequencing to determine trait values

**Figure 1:**
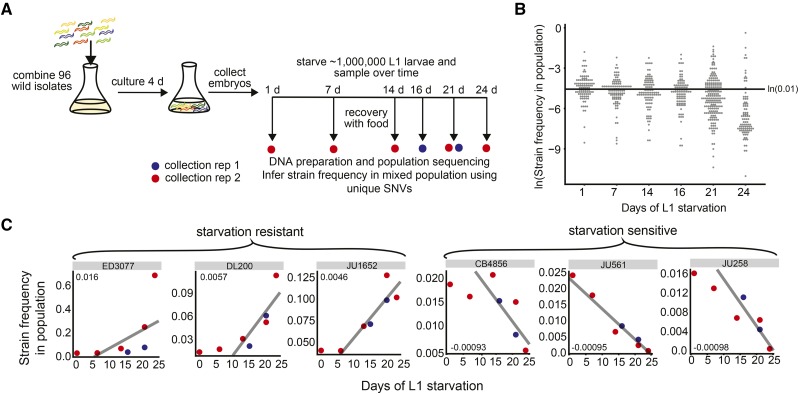
A population selection and sequencing approach to analysis of starvation resistance. A) A schematic of our approach is presented, including pooling of wild strains, co-culture, collection of embryos, establishment of a starvation time series during L1 arrest, and sampling by recovery over time. Time points for each replicate are indicated. B) The natural log of the inferred frequency of all strains in the mixed population over time for both replicates is plotted. The median strain frequency after 1 day of starvation and recovery is ∼1%. C) The inferred frequencies of strains found to have particularly high and low RAD-seq trait values is plotted over time during L1 starvation for replicates 1 and 2 (blue and red, respectively), which included time points spanning early and late starvation. The average regression for replicates 1 and 2 is shown in black, and the RAD-seq trait values (average slope) for each strain are included in the corner of each plot. See Table 1 for RAD-seq trait values for all strains assayed.

We pooled individuals from each of 96 wild isolates that had been genotyped by RAD-seq (Andersen *et al.* 2012) together as larvae, grew them to adulthood in a single large culture, prepared embryos from them, and cultured the embryos in the absence of food so they hatched and entered L1 arrest at controlled density (Figure 1A). At least 7 million L1 larvae were in the starvation culture for each biological replicate, ensuring adequate representation of each strain. We sampled this starved culture over time by taking aliquots and feeding them in separate recovery cultures. Worms exit L1 arrest in the recovery cultures, undergo larval development, and become reproductive. Given the worm density and amount of *E. coli* added, these recovery cultures typically starve out after about one day of egg laying (the exception being deep into starvation). We isolated DNA from recently starved recovery cultures, including parents (the worms recovered from L1 arrest) and their starved L1 progeny (see Materials and Methods). With this sampling approach, we incorporated starvation survival, time to reproduction, and early fecundity, integrating the key facets of starvation resistance to select for haplotypes that retained the greatest fitness following starvation.

Precise measurement of relative strain frequency requires deep sequencing coverage of each strain, making whole-genome sequencing cost-prohibitive. We therefore used RAD-seq as a reduced-representation fractional genome-sequencing strategy (Andrews *et al.* 2016). We used reads including single-nucleotide variants (SNVs) unique to each strain as markers to infer strain frequency. We determined the alternative-to-reference allele ratio for each unique SNV from sequencing reads, with each SNV providing an independent measurement of the frequency of a particular strain in the mixed population. We used the average of these values across all unique SNVs for each strain as the measure of the frequency of the strain in the population. Strains varied in their number of unique SNVs (Table 1), with a median of 33 unique SNVs (25% quartile 22, 75% quartile 67). Consequently, error in frequency estimates in the mixed population presumably varied by strain, with estimates for strains that have more unique SNVs theoretically being more precise (Supplementary Figure 1). The average coverage per unique SNV (independent of allele) also varied, ranging from 812 to 14,878, with a median across all replicates of 1,691 (Table 1). This median level of coverage should theoretically result in less than 5% measurement error if a strain is present at a frequency of 0.01 and has 16 or greater unique SNVs (Supplementary Figure 1). 84 of 96 strains have at least 16 unique SNVs, and in principle all strains made up approximately 1% of the initial pool. However, strains with only 1-2 SNVs (2 strains) do not exhibit 5% or less measurement error unless their frequency approaches 10–15% of the culture. We believe that with these parameters this approach has sufficient power to identify strains that differ significantly in starvation resistance.

We created the 96-strain pool using a single freshly starved plate for each individual strain. As expected, the 96 strains were present in the mixed population at a median frequency of 0.012 on the first day of L1 starvation (Figure 1B). The number of worms per founder plate varies, and the strain pool was cultured for one generation prior to harvesting embryos for L1 starvation, both potentially contributing to variation in strain representation in the pool at the outset of L1 starvation. Thus, there is substantial variation in strain frequency on the first day of starvation (Figure 1B). Critically, we saw a shift in the distribution of strain frequencies, with a clear increase in variance and a decrease in the median over time during starvation. Notably, after 24 days of starvation, the median strain frequency had dropped to 0.00084, and a few strains were present at very high frequency (Figure 1B). Given initial variation in strain frequency, changes in frequency over time were considered. We calculated the slope of a linear regression fit to the strain frequencies over time for each biological replicate and averaged them, generating a “RAD-seq trait value” for each strain (Table 1). The relationship between days of starvation and strain frequency is not necessarily linear for all strains, but this method of determining a trait value provides a simple metric for the degree to which a strain increased or decreased in frequency relative to other strains upon recovery from various durations of starvation. Strains that started at day 1 with low or high relative frequencies could still be classified as resistant, for example, as long as they increased relative to their starting frequency.

**Figure 2:**
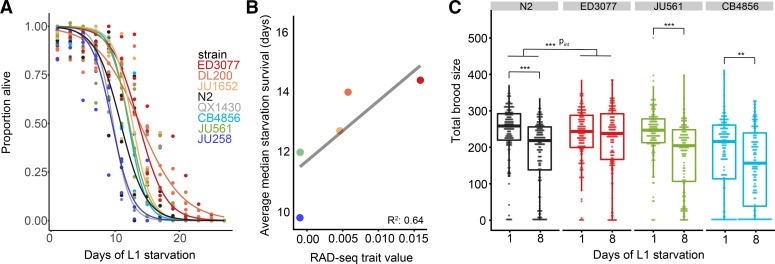
Validation of RAD-seq results with traditional starvation assays. A) L1 starvation-survival curves for strains with the highest and lowest RAD-seq trait values are plotted (see Figure 1C). Starvation survival was assayed for individual strains by manual scoring. Three or four biological replicates were performed, and logistic curves were fit to determine median survival times for each replicate. See Results for individual p-values. B) Correlation between RAD-seq trait value and average median starvation survival is plotted. Multiple R^2^ = 0.64, slope of simple linear regression *P* = 0.055. Note that the point for CB4856 is hidden by the point for JU561. C) Total brood size for N2 (lab reference strain), a strain found to be starvation resistant (ED3077) and a pair of strains found to be relatively starvation sensitive (JU561 and CB4856) is plotted. Each point indicates the total brood size measured for a single worm. Eighteen individual worms were measured per strain, replicate, and days of L1 arrest. Eight biological replicates were scored for a total of 1,152 worms. Linear mixed-effect models were fit for each strain, with days of L1 arrest as a fixed effect and biological replicate as a random effect. To test for an interaction between strain and days of L1 arrest, data from N2 and ED3077 were fit to a linear mixed-effects model with an interaction term; p_int_ = 0.0001. ***P < 0.001, **P < 0.01.

We identified a subset of strains that appeared particularly starvation resistant or sensitive based on RAD-seq trait values (Figure 1C). We wanted to determine 1) whether results obtained from the RAD-seq pooled population estimates correlated well with assays performed on individual strains, and 2) whether the strains of interest affect one or more facets of starvation resistance, since sample collection for RAD-seq integrates multiple facets. We measured both L1 starvation survival over time and brood size following recovery from one or eight days of L1 starvation using traditional assays with individual strains (Figure 2). For L1 starvation survival, we calculated the median survival time for each strain in each replicate after fitting survival curves (Kaplan *et al.* 2015). We found significant differences in the median survival times across all strains (one-way ANOVA, *P* = 0.0049). We were particularly interested in assessing whether starvation-resistant strains differed from starvation-sensitive strains. ED3077 and DL200 increased in frequency over time in the RAD-seq results suggesting relative starvation resistance. These strains exhibited increased starvation survival relative to the standard lab strain, N2, which serves as a reference, though it was not included in the pool because SNVs were identified relative to the N2 reference genome (Figure 2A, *P* = 0.0045 and *P* = 0.0098, respectively). ED3077, DL200, and JU1652 also survived starvation longer than JU258, which displayed one of the largest decreases in frequency in the RAD-seq experiment suggesting relative starvation sensitivity (Figure 2A, *P* = 0.00094, *P* = 0.0021, and *P* = 0.025). As an overall validation of the individual strains assayed, we directly compared RAD-seq trait values with median survival times resulting from the standard starvation-survival assay, and these values were well correlated with an R^2^ of 0.64 (Figure 2B), validating the effectiveness of RAD-seq for identifying particularly starvation-sensitive and resistant strains. There is considerable natural variation in starvation resistance among wild isolates of *C. elegans*, and population selection and sequencing can be used to identify strains that vary for this trait.

To complement the standard L1 starvation survival assay, we measured brood size following one and eight days of L1 starvation to determine total fecundity for a select set of strains (Figure 2C). Fecundity is reduced following eight days of L1 starvation in the N2 reference lab strain (Jobson *et al.* 2015), and early fecundity was integrated into the RAD-seq trait value (see above and Materials and Methods). We hypothesized that a starvation-resistant strain would exhibit less of a starvation-dependent reduction in brood size. We counted the total number of progeny from individual worms for the starvation-resistant strain ED3077, in addition to N2, JU561 and CB4856 (both starvation sensitive in the RAD-seq data). For N2, JU561, and CB4856, worms that were starved for eight days produced significantly fewer progeny than worms starved for only one day (*P* = 1.1 × 10^−8^, *P* = 3.9 × 10^−11^, and *P* = 0.0024, respectively), consistent with a starvation-dependent reduction in fecundity. However, ED3077 did not exhibit a significant difference in brood size depending on the duration of starvation (*P* = 0.30), suggesting a reduced cost of starvation on reproductive success. We explicitly tested for an interaction between genotype and duration of starvation in N2 and ED3077 and found a significant interaction term (*P* = 0.0001), suggesting that ED3077 worms produce more progeny than N2 after experiencing eight days of starvation as L1 larvae, but not one day. These results collectively suggest that ED3077 is buffered against the decrease in reproductive success that typically occurs following eight days of L1 starvation. These results further support the use of RAD-seq on populations to reliably measure differences in life-history traits.

### GWAS of starvation resistance

**Figure 3:**
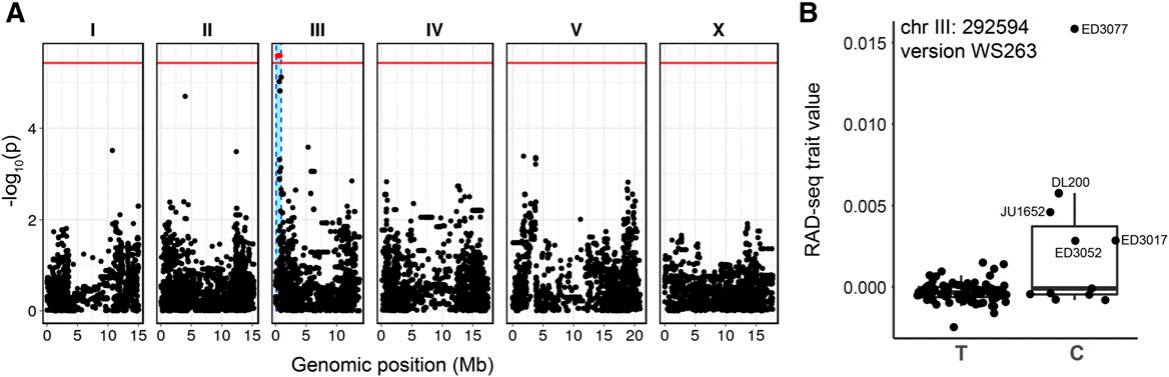
GWAS of RAD-seq trait values identifies a QTL on chromosome III. A) A Manhattan plot for GWAS using RAD-seq trait values (Table 1, Supplementary File 1) as input for 96 strains is presented. The horizontal red line indicates the Bonferroni-corrected threshold for statistical significance at a p-value of 0.05, and is 5.43 for this GWAS. A SNV at chromosome III: 292,584 is significantly associated with starvation survival. B) A genotype-by-phenotype plot for the marker SNV most significantly associated with variation in starvation resistance is presented. Each point represents a particular strain, with its genotype at the SNV and its RAD-seq trait value plotted. The most starvation-resistant strains, and a few others, have the alternative allele (C rather than T).

We used the RAD-seq trait values to determine if observed variation in starvation resistance is significantly associated with genetic variation. We performed GWAS using CeNDR (Cook *et al.* 2017), which implements the EMMA algorithm (Kang *et al.* 2008) to correct for population structure via the rrBLUP package (Endelman 2011). We used RAD-seq trait values for each strain as input. We found that variation in starvation resistance is significantly associated with genetic variation among the strains used (Figure 3, Supplementary File 1, and elegansvariation.org/report/301dcc3f/RADseq_TraitValue). Specifically, a variant located on chromosome III at base pair 292,594 in version WS263 of the *C. elegans* genome was significantly associated with increased starvation resistance. Identification of this association was contingent on using both biological replicates as part of the RAD-seq trait value. By running the EMMA algorithm, CeNDR takes into account linkage disequilibrium, which is prevalent in *C. elegans* (Andersen *et al.* 2012). As a result, the genomic region associated with the trait includes the variant on chromosome III as well as a linked interval ranging from base pair 86,993 to 925,947. This approximately 900 kb region contains 145 protein-coding genes, including 20 variants (in 16 genes) predicted to have high impact on the function of protein-coding genes (Supplementary File 1).

We used crosses to generate a near-isogenic line (NIL) to determine if the associated region on chromosome III affects starvation resistance in a controlled genetic background. Because a NIL contains the genomic region of interest in a different genetic background, the effect of the region on the trait can be directly tested. We introgressed the region on chromosome III from a starvation-sensitive strain into the background of a resistant strain (indicated as strain X > strain Y). We chose strains DL200 and JU258, because DL200 survives significantly longer than JU258 during L1 starvation (Figure 1C, 2A). DL200 also has the alternative SNV for the peak on chromosome III associated with starvation resistance, and JU258 does not (Figure 3B). These strains are theoretically compatible for mating based on known genetic incompatibilities among wild strains of *C. elegans* (Seidel *et al.* 2008; Ben-David *et al.* 2017). We checked the final strain using low-coverage whole genome sequencing. We found that the NIL strain JU258 > DL200 (LRB362) included JU258 sequence on chromosome III from coordinates 1-1,438,286, as expected, as well as a small region on the left end of chromosome I (Supplementary Figure 2). To control for the potential effect of chromosome I region on phenotypes of interest, we also included a RIL in experiments, LRB361, that contains the chromosome III region from DL200 in a majority-JU258 background (Supplementary Figure 2). Like LRB362, LRB361 also retained the chromosome I region from the JU258 background. Despite the retention of the small chromosome I region from JU258 in LRB362, we consider LRB362 a sufficient NIL to test the effect of the chromosome III region of interest on starvation resistance, and we used LRB361 to provide additional support that the chromosome I region is not driving the effect.

**Figure 4:**
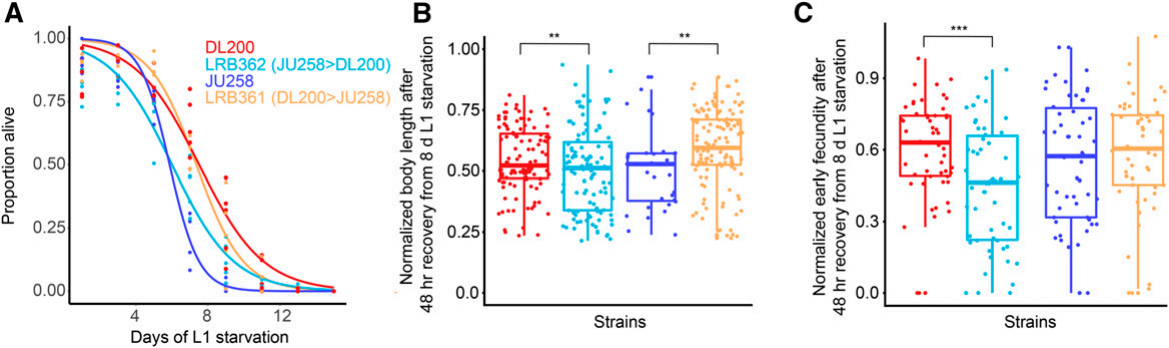
Recombinant strains show that the genomic region on chromosome III associated with variation in starvation resistance affects the trait. A) L1 starvation-survival curves for starvation-resistant strain DL200, starvation-sensitive strain JU258, and newly generated strains LRB361 (DL200 > JU258) and LRB362 (JU258 > DL200). Four to five biological replicates were performed, and logistic curves were fit to determine median survival times for each replicate. See Results for individual p-values. B) Worm length after 48 hr of recovery from eight days of L1 starvation is plotted for the same four strains as in A. Length after eight days of L1 starvation is normalized by length after one day of starvation to isolate the effect of starvation. See Results for individual p-values. C) Brood size on the first day of egg laying (early fecundity; 48 - 72 hr after recovery from starvation by feeding) after recovery from eight days of L1 starvation is plotted. Brood size after eight days of L1 starvation is normalized by brood size after one day of starvation to isolate the effect of starvation on early fecundity. B and C) Each point is an individual worm. Data are pooled from three biological replicates. A linear mixed-effects model was fit to the data using strain as a fixed effect and biological replicate as a random effect. **P < 0.01, ***P < 0.001.

We measured L1 starvation survival in the strains we constructed (LRB361 and LRB362) as well as the parental strains (DL200 and JU258). We found that DL200 survives L1 starvation significantly longer than JU258 (Figure 4A, *P* = 0.00069), validating prior results (Figure 1C, 2A). We were particularly interested in the NIL with the chromosome III region of interest from JU258 in the background of DL200. Median survival of this strain (LRB362) was significantly less than DL200 (*P* = 0.0039). These results suggest that the DL200 genomic region associated with starvation resistance does in fact promote starvation survival, as survival decreases when this region is swapped for the region from JU258. Conversely, median starvation survival of LRB361 is significantly greater than JU258 (*P* = 0.0022). Both LRB361 and LRB362 contain the chromosome I region from the JU258 parent, and LRB362 is starvation sensitive while LRB361 is starvation resistant. We thus find the starvation sensitivity of LRB362 consistent with an effect of the chromosome III region identified in GWAS. These results support the conclusion that the DL200 genomic region on chromosome III associated with increased starvation resistance promotes starvation survival because strains carrying it survive longer than strains that do not. Conversely, strains containing the JU258 genomic region on chromosome III exhibit decreased starvation survival, consistent with starvation sensitivity.

We measured the growth rate and early fecundity of each strain following recovery from one and eight days of L1 starvation to complement analysis of starvation survival. For the RAD-seq experiment, we collected samples after recovery from starvation, integrating effects on survival with effects on growth rate and early fecundity in the final measurement. Thus, measuring all three aspects of starvation resistance is appropriate for validation of the region of interest. To even more closely follow the approach used to generate RAD-seq trait values, we normalized measurements of length (after 48 hr of recovery by feeding) following eight days of L1 starvation by measurements of length following one day of L1 starvation. We expect starvation-sensitive strains to have a larger proportional loss in length following extended starvation than resistant strains. Consistent with our expectation, LRB362 worms were proportionally shorter than DL200 worms after eight days of L1 starvation (Figure 4B, *P* = 0.0076). LRB361 worms were proportionally longer than JU258 worms after eight days of L1 starvation (Figure 4B, *P* = 0.0045). These results further support the conclusion that the region of interest on chromosome III affects starvation resistance, because worms that are more resistant to L1 starvation grow relatively faster upon recovery.

We also counted the number of progeny produced on the first day of egg laying by single worms after recovery from one and eight days of L1 starvation. We again normalized the results after eight days by those after one day to assess the proportional loss in brood size for each strain. Starvation-sensitive strains are expected to produce a proportionally smaller number of progeny after extended starvation. As expected, LRB362 produced proportionally fewer progeny following eight days of starvation than DL200 (*P* = 0.0001), suggesting that the region on chromosome III is important for early fecundity following starvation. Given significant effects on both body length and early fecundity following extended L1 starvation, we conclude that the region of interest on chromosome III contains one or more genetic variants affecting multiple facets of starvation resistance.

## Discussion

We developed a population selection-and-sequencing-based approach to quantitative analysis of starvation resistance in *C. elegans*. We used RAD-seq to infer strain frequency in a pooled population of 96 sequenced wild isolates during selection for starvation resistance. These results together with validation in traditional assays show that substantial natural variation in starvation resistance exists in *C. elegans*, and our results reveal strains that are relatively starvation resistant or sensitive. We identified a region on chromosome III that is associated with variation in starvation resistance, and we used recombinant strains constructed from parental strains DL200 and JU258 to determine that this genomic region from confers starvation resistance. This work demonstrates the feasibility of using sequencing of pooled populations of strains for phenotypic analysis of quantitative traits in *C. elegans*. This approach can be readily applied to a variety of other quantitative traits in *C. elegans* and related nematodes as well as other organisms that can be cultured in sufficiently large numbers. Starvation resistance is likely a fitness-proximal trait in nematodes, and this work establishes a foundation for dissection of this fundamental quantitative trait and identification of genetic variants affecting it.

### Population-selection-and-sequencing-based phenotypic analysis

*C. elegans* are particularly amenable to manipulation in large populations consisting of many strains. Their small size allows millions of individuals to be in a single culture, providing power for quantitative analysis. We used RAD-seq to infer strain frequencies in a population under selection. Though convenient and far more affordable than whole-genome sequencing for quantitative analysis, RAD-seq suffers the limitation that only regions of the genome proximal to *Eco*RI restriction sites are sequenced, the vast majority of which are uninformative for differentiating strains. We chose to focus on unique SNVs to infer strain frequency. However, the number of unique SNVs per strain varies, with a few strains having only one or two unique SNVs. Consequently, sensitivity and precision in measuring strain frequency varies by strain and sequencing depth. The number of unique SNVs depends on the set of strains chosen for pooling. Choosing a subset of highly divergent strains should increase power, as well as increasing sequencing depth at unique SNVs. Future work taking advantage of sequencing methods that target unique SNVs or other strain-specific markers (Mok *et al.* 2017), or sufficiently deep whole-genome sequencing once affordable, should result in substantially improved trait data from such a population-sequencing approach.

Our experimental approach is intended to capture the effects of early-larval (L1) starvation on organismal fitness, incorporating effects on starvation survival, growth, and early fecundity into a single assay. Extended L1 starvation reduces growth rate and fecundity upon recovery in the N2 reference strain (Jobson *et al.* 2015). Furthermore, L1 starvation survival and the ability to grow and develop upon feeding can be uncoupled (Roux *et al.* 2016), and effects of L1 starvation on growth rate and fertility upon recovery can be modified without detectable effects on starvation survival (Hibshman *et al.* 2016). These observations suggest that the most ecologically and evolutionarily relevant way to assess starvation resistance is by assaying the combined effects of starvation on survival, growth rate, and early fecundity. Our experimental method therefore involved taking samples from a starving L1 culture over time and allowing the worms to recover with food. If worms were dead, they did not recover and contributed little if any DNA to the sample. However, if they survived but were relatively slow to become reproductive adults, or if their fecundity was compromised, then their representation was reduced in the DNA that was sequenced. Importantly, because we measured recovery on the first day of starvation, before effects on growth rate or fecundity are detectable (Jobson *et al*. 2015), as well as late time points, strains that simply developed faster or were more fecund in our culture conditions were not considered starvation resistant. That is, starvation-resistant strains were identified as those strains with frequencies that increased over time during starvation, as captured by the RAD-seq trait value. Importantly, we controlled for potential intergenerational or transgenerational effects (Jobson *et al.* 2015; Hibshman *et al.* 2016; Webster *et al.* 2018; Jordan *et al.* 2019) by maintaining wild strains as well fed prior to experiments for at least three generations, and starvation during the experiment was experienced by all strains in parallel. We also controlled for worm density during starvation. Critically, we validated the RAD-seq results with traditional, manual starvation assays. Alternative sampling strategies in future studies could be used to isolate different effects of starvation, potentially identifying genetic variants that affect specific aspects of starvation resistance (*e.g.*, mortality, growth rate or fecundity). Various life-history traits could also be analyzed independent of starvation.

It is important to consider that by sequencing populations we measured relative as opposed to absolute starvation resistance. ED3077 and DL200 increased in frequency over time, suggesting increased starvation resistance relative to other strains. However, strains that decreased in frequency over time are not necessarily starvation sensitive in absolute terms – they are sensitive relative to other strains in this pooled context. Furthermore, relative survival may depend on the composition of the population. Population density affects L1 starvation survival in *C. elegans* (Artyukhin *et al.* 2013). There may be complex effects due to competition for resources or natural variation in production of or sensitivity to pheromones or excreted metabolites. While we included two biological replicates in our analysis, additional replication would be helpful for determining how often strains exhibit the same directionality and magnitude of change over time. Future studies could determine the effects of population composition on population dynamics during starvation, and in particular if there is natural variation in density-dependence of starvation survival.

### Identification of a genomic interval affecting starvation resistance in wild isolates of C. elegans

*C. elegans* displays substantial phenotypic plasticity in response to nutrient availability, similar to other nematodes and many animals in general (Fusco and Minelli 2010; Viney and Diaz 2012). *C. elegans* survives on ephemeral food supplies and thrives with boom-and-bust population dynamics (Schulenburg and Felix 2017). Their ability to adapt to starvation, survive, recover, and reproduce is important for fitness and therefore likely to be under relatively strong selection. As a result, it was unclear at the outset of this study how much natural variation in starvation resistance would be identified. Our results suggest that there is substantial natural variation in starvation resistance in *C. elegans*.

We took advantage of the extensive genetic and genomic resources available in *C. elegans* to perform a statistical genetic analysis of starvation resistance. Many *C. elegans* wild isolates have had their genomes sequenced with variants called (Cook *et al.* 2017). Population genetic analyses have been performed, and relatedness among individual strains is known. We used CeNDR to perform GWAS to determine if any regions of the genome are associated with starvation resistance using RAD-seq trait values from 96 strains. One might imagine that the trait is so polygenic or epistatic that genetic variants that influence it may be numerous and of small effect, making them difficult to identify. However, a 900 kb region on the left side of chromosome III was found to be associated with the trait. Efforts to map starvation resistance in *Drosophila* have identified multiple genomic loci, demonstrating the polygenicity of the trait in another model system (Everman *et al.* 2019). The genomic interval we identified explains ∼2% of the observed variation in starvation resistance. Additional studies will likely identify additional loci to further explain this presumably polygenic trait.

We generated a NIL that contained the region of interest from a starvation-sensitive strain, JU258, introgressed into the background of a starvation-resistant strain, DL200, to test functional significance of this genomic region. Despite containing ∼98% of the genome of DL200, this NIL survived L1 starvation significantly less than DL200, and survival did not differ significantly from JU258. This suggests that this region is important for conferring starvation resistance in DL200. Consistent with this result, the survival of a RIL that contained the chromosome III region from DL200 but also contained a majority of the JU258 genetic background was not statistically different from DL200. Analysis of growth rate and early fecundity following eight days of starvation also support the conclusion that this genomic region affects starvation resistance. Though we showed this region on the left side of chromosome III is important for starvation resistance, it is unknown whether there is one particular variant that confers resistance or whether there are multiple causal variants within the region.

### Future directions for genetic analysis of starvation resistance and other quantitative traits

The genetic architecture of starvation resistance in *C. elegans* remains to be determined. New strains are being cataloged and sequenced regularly (Crombie *et al.* 2019), adding to the genetic diversity of *C. elegans* and providing additional leverage for analysis of starvation resistance and other traits. Use of more divergent strains could facilitate identification of multiple QTL. Additional annotation of genetic variation beyond SNVs, and *de novo* assembly of particularly divergent strains, should also increase the effectiveness of association mapping.

Population selection and sequencing of wild isolates accelerates measurement of quantitative trait values across panels of isogenic wild strains. These panels can be used for statistical genetic approaches, but linkage disequilibrium poses a potential drawback, especially in *C. elegans* (Andersen *et al.* 2012), as seen in the large size of the QTL we identified. As a complementary approach, genetic recombination can be used to disrupt linkage disequilibrium. Recombinant strain panels such as the Collaborative Mouse Cross, Drosophila Synthetic Population Resource, and the *C. elegans* Multiparental Experimental Evolution Panels have been generated through crosses of divergent strains (Churchill *et al.* 2004; Mackay *et al.* 2012; Noble *et al.* 2017). A number of multiparental panels have also been generated for *Arabidopsis*, maize, wheat, rice, and other plants (Buckler *et al.* 2009; Mcmullen *et al.* 2009; Bandillo *et al.* 2013; Mackay *et al.* 2014; Thépot *et al.* 2015; Bouchet *et al.* 2017; Kidane *et al.* 2019). These panels enable relatively fine mapping by disrupting linkage disequilibrium. Similarly, RILs can be generated from just two parental strains in order to narrow down causal genomic intervals. Approaches to quickly generate large numbers of recombinant individuals in a single population for bulked-segregant analysis (BSA) have been effective for fine mapping in yeast (Ehrenreich *et al.* 2010) and, more recently, in *C. elegans* (Burga *et al.* 2019). However, multiparental lines, RILs, and BSA are limited to the genetic variation of the parents included in the crossing scheme, and genetic variation can be lost due to selection or drift if sufficiently large population sizes are not maintained (Rockman and Kruglyak 2009; Baldwin-Brown *et al.* 2014). In order to identify which parents will be most informative for dissecting a particular trait, and to ascertain the phenotypic variation of a trait in the wild, it is of interest to first assay many wild strains. We therefore see population selection and sequencing of isogenic wild strains as a valuable complement to these existing approaches. In addition, multiparental and RIL panels can also potentially be pooled and subjected to population selection and sequencing. Population-selection-and-sequencing-based approaches together with ever-richer genomic resources should provide the power to dissect a variety of complex quantitative traits in *C. elegans* and related nematodes.
